# Identification of *Xylaria cubensis* Isolated from *Moringa oleifera* Leaves and Its Antibacterial Potentials

**DOI:** 10.21315/tlsr2026.37.3.9

**Published:** 2026-10-15

**Authors:** Siti Aisyah Daud, Wong Chaim Tung, Latiffah Zakaria, Nurul Farizah Azuddin, Tan Wen Nee, Nurhaida, Syarifah Ab Rashid, Yee Simone

**Affiliations:** 1 School of Biological Sciences, Universiti Sains Malaysia, 11800 Pulau Pinang, Malaysia; 2 School of Distance Education, Universiti Sains Malaysia, 11800 Pulau Pinang, Malaysia; 3 Department of Chemistry, Faculty of Mathematics and Natural Sciences, Syiah Kuala University, Aceh, Indonesia

**Keywords:** Endophytic Fungi, *Moringa oleifera*, Antibacterial, *Bacillus subtilis*, Volatile Compounds, Kulat Endofit, Moringa oleifera, Antibakteria, Bacillus subtilis, Sebatian Meruap

## Abstract

The escalating threat of antimicrobial resistance underscores the need for novel natural therapeutic agents. Endophytic fungi are recognised as prolific producers of bioactive metabolites with antimicrobial potential. In this study, an endophytic fungus isolated from mature leaves of *Moringa oleifera* was molecularly identified as closely related to *Xylaria cubensis*. Its ethyl acetate extract exhibited notable antibacterial activity against *Bacillus subtilis*, with a minimum inhibitory concentration (MIC) of 25 mg/mL and a minimum bactericidal concentration (MBC) of 100 mg/mL. Scanning electron microscopy revealed pronounced morphological alterations in treated B. subtilis cells, including shrinkage and lysis, consistent with membrane disruption. Gas chromatography–mass spectrometry (GC–MS) analysis identified four major compounds: 2,4-di-tert-butylphenol, pyrrolo[1,2-a]pyrazine-1,4-dione (hexahydro-3-(1-methylethyl)), 3-furanacetic acid (4-hexyl-2,5-dihydro-2,5-dioxo-) and 1,3,5-xylenol. These findings highlight the antibacterial potential of endophytic *X. cubensis* from *M. oleifera*, particularly against *B. subtilis*, and support the role of endophytic fungi as promising sources of natural antimicrobial compounds for future therapeutic development.

## Abstrak

Ancaman rintangan antimikrob yang semakin meningkat menekankan keperluan untuk agen terapeutik semula jadi baharu. Kulat endofit dikenali sebagai pengeluar metabolit bioaktif dengan potensi antimikrob yang tinggi. Dalam kajian ini, satu kulat endofit yang diasingkan daripada daun matang *Moringa oleifera* telah dikenal pasti secara molekul sebagai berkait rapat dengan *Xylaria cubensis*. Ekstrak etil asetat menunjukkan aktiviti antibakteria yang ketara terhadap *Bacillus subtilis*, dengan kepekatan perencatan minimum (MIC) sebanyak 25 mg/mL dan kepekatan bakterisid minimum (MBC) sebanyak 100 mg/mL. Analisis mikroskop elektron imbasan (SEM) mendedahkan perubahan morfologi yang jelas pada sel *B. subtilis* yang dirawat, termasuk pengecutan dan lisis sel, konsisten dengan gangguan membran. Analisis kromatografi gas–spektrometri jisim (GC–MS) mengenal pasti empat sebatian utama: 2,4-di-tert-butylphenol; pyrrolo[1,2-a]pyrazine-1,4-dione (hexahydro-3-(1-methylethyl)); 3-furanacetic acid (4-hexyl-2,5-dihydro-2,5-dioxo-); dan 1,3,5-xylenol. Penemuan ini menonjolkan potensi antibakteria *X. cubensis* endofit daripada *M. oleifera*, khususnya terhadap *B. subtilis*, serta menyokong peranan kulat endofit sebagai sumber semula jadi yang menjanjikan untuk pembangunan terapeutik masa depan.

**HIGHLIGHTS**

- An endophytic fungus isolated from *Moringa oleifera* leaves was identified as closely related to *Xylaria cubensis*.
- The ethyl acetate extract showed selective antibacterial activity against *Bacillus subtilis* and *B. cereus*.
- The extract showed bactericidal activity against *B. subtilis* and caused membrane damage and cell lysis.
- This is the first report of 2,4-di-tert-butylphenol production by *Xylaria cubensis*, highlighting its antibacterial potential.

## INTRODUCTION

The increasing use and misuse of antibiotics and other antimicrobial drugs have accelerated the emergence of multidrug-resistant (MDR) pathogens, significantly reducing the effectiveness of existing therapies. The rapid dissemination of these MDR pathogens now constitutes a major global health challenge, leading to higher morbidity and mortality. The World Health Organisation (WHO) maintains a global priority pathogens list to guide the research and development of new antimicrobial agents. The most recent 2024 list categorises bacteria based on the urgency for new treatments:

1. Priority 1 (Critical) includes carbapenem-resistant Enterobacterales, third-generation cephalosporin-resistant Enterobacterales, carbapenem-resistant *Acinetobacter baumannii* and rifampicin-resistant *Mycobacterium tuberculosis*.
2. Priority 2 (High) includes fluoroquinolone-resistant *Salmonella Typhi*, vancomycin-resistant *Enterococcus faecium*, carbapenem-resistant *Pseudomonas aeruginosa* and methicillin-resistant *Staphylococcus aureus* MRSA.
3. Priority 3 (Medium) includes macrolide-resistant *Streptococcus pneumoniae*, penicillin-resistant Group B Streptococci and ampicillin-resistant *Haemophilus influenzae* (World Health Organisation 2024).

Despite the urgent need for novel antimicrobials, the current antibiotic development pipeline remains limited. First-line antibiotics are generally preferred due to their high efficacy and lower toxicity; however, resistance to these agents is rising. This issue extends beyond the WHO-listed pathogens to unlisted bacteria such as *Bacillus* species. *Bacillus* strains have been reported to exhibit complete resistance to commonly used first-line antibiotics, including penicillin, vancomycin and amoxicillin, highlighting the critical need for new therapeutic strategies (Thanganadar *et al*. 2021).

*Moringa oleifera* (MO), commonly referred to as horseradish tree are known globally for its medicinal advantages. The tree is a highly beneficial herbal plant from family Moringaceae, which is also known as the ‘miracle tree’ due to its high levels nutrients and numerous therapeutic properties (Dhakad *et al*. 2019). Various studies demonstrated the antioxidant, anti-inflammatory, anti-cancer, and antimicrobial capabilities of the *Moringa* tree and its extract (Hodas *et al*. 2021; Islam *et al*. 2021; Khan *et al*. 2017). Moreover, MO also reported to have the ability to produce an insulin-like substance that aids in lowering blood glucose level, thus serving as a potential alternative for diabetes treatment (Paula *et al*. 2017). Besides, research also outlined the antimicrobial activities by MO due to the presence of beneficial compounds such as bistortaside A. quercetin and decanoic acid which exhibit the inhibitory activity against pathogens including MRSA, *B. subtilis* and *C. albicans* (Devan *et al*. 2019; YounisKhalaf *et al*. 2021).

Significantly, the *Moringa* tree harbors various endophytes, including fungi and bacteria within its leaves (Ekpunobi & Enyi 2022). Endophytes are the colonisers of the internal plant tissues, which inhabit without causing any disease or hostile symptoms and can be isolated from surface-sterilised plant tissues. Interestingly, endophytic microorganisms such as fungi have been reported to produce a wide range of secondary metabolites that mimic those of their host plants under *in vitro* conditions, thereby reducing dependence on endangered plant species for metabolite extraction (Rani *et al*. 2022). Moreover, studies have revealed that bioactive compounds isolated from crude extract of endophytic fungi, such as ophiobolins and pestaloficiols, exhibit antibacterial activity against the aforementioned pathogens and cytotoxic activity against the human cell line HeLa, respectively (Wen *et al*. 2022). Thus, the presence of the endophytic fungi and their symbiotic interaction with host plants such *M. oleifera* may enhance the likelihood of discovering alternative bioactive compounds with therapeutic potential while aiding an environmentally friendly approach in exploring beneficial secondary metabolites. The present study aimed to isolate and identify an endophytic fungus from *M. oleifera* leaves and to evaluate the antibacterial activity of its extracts against Gram-negative and Gram-positive bacteria as well as selected yeasts. In addition, the volatile antibacterial compounds in the extracts were characterised using GC–MS analysis.

## METHODOLOGY

### Test Microorganisms

*Bacillus cereus, B. subtilis, Escherichia coli, Pseudomonas aeruginosa, Salmonella typhimurium, Staphylococcus aureus* and *Candida albicans* were from the culture stock of Laboratory 318, School of Biological Science, Universiti Sains Malaysia. The bacteria and yeast were sub-cultured on the fresh nutrient agar (NA; HiMedia, India) plate and potato dextrose agar (PDA; Himedia, India) plates, respectively. Bacterial plates were incubated at 37°C, while yeast plates were incubated at 30°C. Following incubation, all plates were stored at 4°C for subsequent use.

### Plant Sampling, Isolation and Validation of Endophytic Fungi from *M. oleifera* Leaves

Healthy, mature *M. oleifera* leaves without visible disease symptoms were collected from plants free of fungicide and pesticide applications. The samples were placed in sterile zipper plastic bags and processed on the day of collection. Surface sterilisation was performed following the method of Sahani and Thakur (2017). Briefly, leaf samples were sequentially immersed in 70% (v/v) ethanol and 5% (v/v) sodium hypochlorite for 2 min each, followed by a final immersion in 70% (v/v) ethanol for 1 min. The samples were rinsed twice with sterile distilled water and air-dried under sterile conditions. Sterilised leaves were cut into approximately 5 mm × 5 mm segments. The efficacy of surface sterilisation was verified using the surface imprinting technique described by Anjum and Chandra (2015) by pressing both sides of each segment onto potato dextrose agar (PDA) supplemented with chloramphenicol (50 mg/mL). In addition, 100 μL of the final rinse water was spread onto PDA plates and incubated at 30°C for 5 days to confirm the absence of epiphytic microorganisms. The leaf segments were then placed onto PDA supplemented with chloramphenicol and incubated at 30°C for 15 days. Emerging fungal hyphae were sub-cultured onto fresh PDA plates to obtain pure endophytic fungal isolates. Pure cultures exhibiting distinct colony morphologies were selected for further analysis, sub-cultured biweekly, and stored at 4°C for subsequent studies. These isolates were designated *M. oleifera* leaf 1 (MOLEF1) to *M. oleifera* leaf 7 (MOLEF7) to differentiate their original sources.

### Agar Plug Assay

The assay was conducted as the preliminary screening of isolates’ antimicrobial activity and was performed on the Mueller-Hinton agar (MHA; HiMedia, India) plates. A sterile cotton swab impregnated with microbial suspension (0.5 M McFarland) was swabbed evenly onto the fresh MHA plate. Agar plugs (approximately 6 mm in diameter) of the endophytic fungi which was previously cultured on PDA plate were obtained using cork borer and placed onto pre-inoculated PDA plate facing downward (Sahani & Thakur 2017).

### Identification of Endophytic Fungus

Identification was conducted through macroscopic, microscopic, and molecular analyses. Macroscopic characteristics, including colony surface texture, margins and pigmentation, were evaluated on potato dextrose agar (PDA) (Dos Reis *et al*. 2022). Microscopic structures were examined using both light microscopy and scanning electron microscopy (SEM) (Woei *et al*. 2024). Fungal isolates were molecularly identified using 18S rDNA sequencing. Mycelia were cultured in potato dextrose broth (PDB; HiMedia, India) at 30°C for 7 days, harvested, frozen in liquid nitrogen, and ground into a fine powder. Genomic DNA was extracted from ~60 mg of fungal tissue using the Invisorb® Spin Plant Mini Kit (Stratec, Germany) following the manufacturer’s protocol. The β-tubulin gene was amplified by PCR using primers T1 and T22 in a 50 μL reaction volume on a Bio-Rad MyCycler™ Thermal Cycler. PCR conditions consisted of initial denaturation at 95°C for 5 min, followed by 35 cycles of 94°C for 30 s, 52°C for 40 s and 72°C for 1 min, with a final extension at 72°C for 10 min. Amplicons were verified on 1% agarose gel and sequenced by NHK Bioscience Solutions (Malaysia).

### Fermentation and Extraction of Fungal Secondary Metabolites

The method described by Woei *et al*. (2024) was applied in this section. The yeast extract sucrose (YES) broth consisted of 0.40% (w/v) yeast extract (HiMedia, India), 2% (w/v) sucrose (VWR International, USA), 0.10% (w/v) potassium dihydrogen phosphate (KH_2_PO_4_, Chemiz, Malaysia) and 0.05% (w/v) magnesium sulfate (MgSO_4_, System Chemicals, Malaysia). The medium was calibrated to pH 5.50 using 1M HCl and 1M NaOH and sterilised via autoclaving at 121°C for 15 min. A total of 6 pieces of fungal agar plugs (6 mm in diameter) excised from pure culture on PDA medium were cultured in 100 mL yeast extract sucrose (YES) broth within 250 mL conical flasks and incubated at room temperature (28 ± 2°C) for 15 days. After the incubation, the fermentation products were filtered through a Muslin cloth and subsequently extracted three times with equal volume (1:1; v/v) of ethyl acetate (Qrec, New Zealand) using liquid-liquid extraction method. The metabolites product was concentrated by evaporating under reduced pressure using a rotary evaporator at 50°C to 60°C. Then, the crude extract was dried at 40°C in the oven and later stored at 4°C for further study.

### Antimicrobial Activity Screening

#### Well diffusion assay

The assay was based on the method described by Omar *et al*. (2019) with a slight modification. A bacterial suspension (0.5 McFarland) was evenly spread on the fresh Mueller Hinton agar (MHA; HiMedia, India) plate, and 7 mm agar wells were impregnated with 20 μL of extract. The crude extract was dissolved in methanol (Qrec, New Zealand) and diluted with sterile distilled water to 25% methanol concentration, maintaining at 20 mg/mL extract concentration. The respective wells also pipetted with 20 μL of 25% (v/v) methanol which served as negative control and chloramphenicol (30 mg/mL) as positive control. The plates were incubated at 37°C for 24 h and the formation of clear zones was measured in millimeters (mm).

#### Broth microdilution assay

The assay was conducted following the protocol by Rozman *et al*. (2020) to determine the minimum inhibitory concentration (MIC) of the fungal extract, the lowest concentration of antimicrobial agent preventing visible microbial growth. The test microorganisms with significant inhibitory activity in well diffusion assay was chosen. The assay was carried out in sterile 96-well plate using double-strength nutrient broth to prepare microbial inocula which were diluted to 10^5^ CFU/mL. The fungal extract was diluted accordingly to a concentration raging between 0.78 to 100 mg/mL. Each well containing 100 μL of microbial inoculum and 100 μL of fungal extract. Meanwhile, the control group wells containing 25% methanol, 30 mg/mL chloramphenicol for negative and positive control respectively and another two controls for sterility and growth. After the incubation at 37°C for 24 h, 40 μL of p-iodonitrotetrazolium violet salt (INT salt, Macklin, China) was added and the colour change from yellow to violet which indicate the microbial growth was observed. The MIC value was determined based on the lowest concentration with no colour changes. Subsequently, the minimum bactericidal concentration (MBC) was determined by streaking aliquots from each well into fresh nutrient agar plates, followed by incubation at 37°C for 24 h. The MBC was defined as the lowest extract concentration that resulted in the complete absence of microbial growth.

### SEM Observation of Cellular Degeneration

The fungal extract was prepared at a final concentration of 100 mg/mL. Subsequently, 1 mL of the extract was added to flasks containing overnight bacterial inocula to a final volume of 20 mL, while control flasks received 1 mL of sample diluent (25% methanol). All flasks were incubated at 37°C for 20 h under shaking conditions at 150 rpm. After incubation, bacterial cells were harvested by centrifugation at 1,500 × g for 10 min and fixed in McDowell-Trump fixative, then stored at 4°C. Preparation of the cell pellet for SEM analysis followed the HDMS (hexamethyldisilazane) method. The fixed pellet was washed with phosphate buffer and treated with 1% osmium tetroxide (OsO_4_) for 1 h. Samples were then washed with distilled water and dehydrated through a graded ethanol series (50%, 75%, 90% and 100%) with 10 min intervals at each concentration. The dehydrated samples were treated with hexamethyldisilazane for 10 min and air-dried in a desiccator. Finally, the dried cells were mounted onto specimen holders, coated with gold-palladium and observed under a scanning electron microscope (Hitachi S-4700 FESEM, Japan) (Woei *et al*. 2024).

### Identification of Volatile Compound from Fungal Extract

One milligram of crude extract paste was dissolved in high-purity methanol (≥ 99.8%) and injected into a Hewlett-Packard 6890 Network gas chromatography system (Rozman *et al*. 2020). The analysis was performed using an HP-5MS column (Agilent, USA) coupled with a Hewlett-Packard 5973 inert mass selective detector for compound identification. The GC oven was initially heated to 70°C for 2 min, ramped to 280°C over 30 min, and maintained at 280°C for 45 min. Helium was used as the carrier gas at a constant flow rate of 1.0 mL/min. Compounds were identified by comparing their mass spectra and chromatograms with the NIST17 database.

### Statistical Analysis

Data from the well diffusion assay and broth microdilution were obtained in triplicate. Results from the well diffusion assay were analyzed using one-way ANOVA followed by Tukey’s post hoc test (SPSS Version 27, IBM, USA).

## RESULTS

### Isolation and Identification of the Isolated Endophytic Fungi and Antimicrobial Screening

The agar plug assay was employed to evaluate the antimicrobial potential of the fungal isolates. The activities of six isolates against seven test microorganisms are summarised in Table 1. Overall, the antimicrobial spectrum varied among the isolates. MOLEF 1, MOLEF 2 and MOLEF 5 exhibited inhibitory activity only against *S. typhi*, whereas MOLEF 3 showed no detectable inhibitory effect. MOLEF 6 inhibited *S. aureus* and *P. aeruginosa*, while MOLEF 7 inhibited these bacteria in addition to *B. cereus* and *E. coli*. Notably, MOLEF 4 demonstrated the broadest antimicrobial activity, inhibiting all tested bacterial species except the yeast *C. albicans*. Consequently, MOLEF 4 was selected for molecular identification and subsequent studies.

The characterisation of the endophytic fungus MOLEF 4 was achieved by adapting both macroscopic and microscopic observation. The macroscopic characteristics of isolate MOLEF 4 are shown in Fig. 1. The isolate exhibited progressive radial expansion and mycelial densification from day 7 to day 30 of incubation. On day 7, colonies appeared circular with uniform, white, cottony aerial mycelia and smooth margins (Fig. 1A). By day 15, increased milky white mycelial density and radial growth were observed, accompanied by subtle surface textural differentiation (Fig. 1B). The fungal culture initially exhibited a cottony texture with entire margins and a zonate pattern characterized by concentric rings, followed by the development of prominent brownish stromatic structures by day 30 (Fig. 1C). The microscopic observation of the fungus sample was carried out using a light microscope and a scanning electron microscope (SEM). The study revealed that MOLEF 4 consisted of septate hyphae, and a slender, smooth conidiophore-like structure arose from the mycelia (Fig. 2). Microscopic examination of isolate MOLEF 4 under magnification of 4× revealed a dense, intertwined hyphal network with irregular branching patterns (Fig. 2A). At a higher magnification (40×), a localised hyphal swelling at specific regions was observed (Fig. 2B). This swollen hyphal structure was further confirmed by scanning electron microscopy, which demonstrated a compact mycelial architecture with distinct swollen and occasionally deformed hyphae (Fig. 2C). Such swollen hyphal features are commonly reported in *Xylaria* sp., suggesting that MOLEF 4 may belong to the genus *Xylaria*. Based on these morphological characteristics, MOLEF 4 was therefore selected for molecular identification to confirm its taxonomic placement.

Purified DNA from the β-tubulin region of MOLEF 4 was amplified by PCR, and the product was confirmed by gel electrophoresis, which showed a band of approximately 1,500 bp (Fig. 3). The DNA sequence obtained was analysed using BLAST, and the top 10 homologous sequences are presented in Fig. 4. The results indicated that MOLEF 4 is closely related to *Xylaria cubensis*, showing 96.04% sequence similarity.[Table t2-tlsr-37-3-181]

### Antibacterial Activity and Structural Degradation of Treated *B.Subtilis* Cells

The antibacterial activity of MOLEF 4 was evaluated against seven pathogenic strains using well diffusion and broth microdilution assays. In the well diffusion assay, MOLEF 4 exhibited antibacterial activity only against *Bacillus* species (Fig. 5). Inhibition zones of approximately 10 mm were observed for both *B. cereus* (Fig. 5A) and *B. subtilis* (Fig. 5B). The MIC was determined using a 96-well plate assay, where a colour change from yellow to violet indicated bacterial growth. The values were 25 mg/mL for *B.subtilis* and 12.5 mg/mL for *B. cereus*, highlighting the extract’s selectiveefficacy. The MBC was also assessed, with no bacterial growth observedat 100 mg/mL for either strain, confirming the extract’s bactericidalpotential against these pathogens. The resulting MBC/MIC ratios (4 for *B.subtilis* and 8 for *B. cereus*) indicate that the extract exhibits bactericidalactivity against *B. subtilis* and a bacteriostatic effect against *B. cereus*. Based on these results, *B. subtilis* was considered the most susceptible and selected for further cellular degradation analysis. SEM examination revealed clear differences in cellular morphology between untreated and extract-treated cells (Fig. 6). The untreated control cells appeared healthy and were larger than the treated cells (Fig. 6A). In contrast, most cells exposed to the extract were smaller and shrunken, with some exhibiting lysis and damaged or perforated cell membranes, although a few cells remained intact (Fig. 6B). These observations indicate that the crude extract of MOLEF 4 is capable of disrupting the cellular structure of *B. subtilis*.

### Detection of Volatile Compounds via GC-MS Analysis

GC–MS analysis was conducted to identify volatile constituents of the fungal extract. In total, 42 compounds were detected, but only four major compounds had normalised area values exceeding 10% based on comparison with the NIST 2017 library. As illustrated in Fig. 7 and summarised in Table 3, the major constituents were 2,4-DTBP; pyrrolo [1,2-a]pyrazine-1,4-dione, hexahydro-3-(1-methylethyl); 3-furanacetic acid, 4-hexyl-2,5-dihydro-2,5-dioxo-; and 1,3,5-xylenol. Among these, 2,4-DTBP had the highest normalised area (19.26%), followed by pyrrolo[1,2-a]pyrazine-1,4-dione, hexahydro-3-(1-methylethyl) (16.93%), 3-furanacetic acid, 4-hexyl-2,5-dihydro-2,5-dioxo- (10.58%) and 1,3,5-xylenol (10.29%).

## DISCUSSION

In recent years, endophytes isolated from various plants, particularly medicinal species, have been reported to produce secondary metabolites with potent antimicrobial activities. In the present study, mature leaves of *M.oleifera* were used as the source for isolating endophytic fungi, based onthe well-documented therapeutic properties of *M. oleifera* leaf extracts. Previous work by Fan *et al*. (2020) demonstrated that endophytic fungaldiversity can vary with leaf age, with younger leaves generally harboringa greater abundance and diversity of endophytes. Previous studies hadsuccessfully isolated endophytic fungi from *M. oleifera* leaves frommultiples genera including *Fusarium*, *Xylaria*, *Pestalotiopsis*, *Aspergillus*, *Nigrospora*, *Stachybotrys*, *Rhizoctonia*, *Macrophomina*, *Cladosporium* and *Colletotrichum* (Atri *et al*. 2020; Carbungco *et al*. 2017).

Contradictory results were obtained between the agar plug and well diffusion assays. In general, the agar plug assay utilises solid-state antimicrobial compounds produced directly by the fungal colony, whereas the well diffusion assay relies on liquid extracts. This difference can lead to the loss of bioactive compounds that are poorly extractable or the degradation of unstable compounds under incubation conditions. However, this limitation can also be advantageous, as the well diffusion assay may eliminate problematic compounds, saving time and resources (Rütten *et al*., 2022). A similar effect was observed during the application of the MOLEF 4 ethyl acetate extract against *Bacillus* species, suggesting that the extracted compounds are active against these bacteria, while other potential antimicrobial metabolites effective against different pathogens may not have been recovered using this solvent. As noted by Pauzi *et al*. (2021), agar diffusion methods may not fully reflect the true antimicrobial potential, as results can be influenced by the solubility and diffusibility of the sample, highlighting the need for further evaluation.

Identification of the genus or species of MOLEF 4 is important for understanding its biological characteristics and previously reported antimicrobial potential, thereby providing context for the observed effects of its ethyl acetate extract against pathogenic microorganisms. Members of the family Xylariaceae are known to produce distinctive finger-like stromatic structures during growth (Sica *et al*. 2016). Similarly, Wu *et al*. (2022) described morphological features characteristic of *X.cubensis*, including the development of small brown stromata on culturemedia, which are comparable to the observations made for MOLEF 4 in the present study. Although Wu *et al*. (2022) reported the absence of conidia in *X. cubensis* cultures, an earlier study by Rogers and Samuels(1986) documented the presence of conidia and conidiophores in this species.

Consistent with these earlier findings, microscopic examination of MOLEF 4 revealed the presence of conidiophore-like and conidia-like structures. These structures may correspond to swollen hyphae or meristem arthroconidia, which are specialised asexual fungal spores formed in basipetal chains, with the youngest cells located at the base, arising from a meristematic apex of the conidiophore. This evidence further supports the classification of MOLEF 4 within the genus *Xylaria*. Based on BLAST analysis, MOLEF 4 was found to be closely related to *X. cubensis*. Endophytic species of *Xylaria* have previously been reported among fungi isolated from *M. oleifera* leaves (Carbungco *et al*. 2017). Furthermore, Jha *et al*. (2023) reported that several *Xylaria* species, including *X. grammica*, *X. mali* and *X. cubensis*, are known to produce bioactive secondary metabolites, which exhibit broad-spectrum antimicrobial and other therapeutic activities.

Microbial growth in the sample treatments was assessed using a colourimetric analysis. INT (iodonitrotetrazolium) salt serves as an indicator of living microorganisms in a 96-well plate. The colorless tetrazolium salt functions as an electron acceptor and is reduced by metabolically active cells to a red-coloured formazan. The MIC was defined as the lowest extract concentration that inhibited growth, indicated by a reduction in colour intensity or the absence of the red formazan product (Mahdi *et al*. 2014). Lower MIC values indicate higher antimicrobial efficacy, as less extract is required to inhibit susceptible microorganisms. According to Table 1, *B. subtilis* was more susceptible to the crude extract from MOLEF 4 than *B. cereus*. The difference in susceptibility may be attributed to several factors, including variations in cell wall composition (Malanovic & Lohner 2016), differences in surface hydrophilicity and negative charge distribution (Ma *et al*. 2017), production of enzymes capable of degrading antimicrobial agents (Luthra *et al*. 2018), and the ability to synthesise exopolysaccharides (EPS), which can impede antibiotic diffusion (Kumar Singha 2012).

Antibiotics can function as either bacteriostatic agents, which inhibit bacterial growth or bactericidal agents, which actively kill bacteria. Bactericidal agents are often preferred for more effective infection control, as bacteriostatic drugs typically require a fully functioning host immune system to eradicate bacterial growth (Jung *et al*. 2022). However, bacteriostatic drugs may be associated with a lower risk of toxic shock and generally have more tolerable side-effect profiles (Loree & Lappin 2023). Despite these advantages, bactericidal antibiotics may lead to bacterial regrowth once the drug is removed, potentially contributing to the development of drug resistance (Levin *et al*. 2017). Therefore, determination of MIC and MBC is a crucial step in evaluating the overall efficacy of an antimicrobial agent. According to Mogana *et al*. (2020), a drug is considered bactericidal if the MBC/MIC ratio is ≤ 4, while a ratio exceeding 4 is classified as bacteriostatic. MBC values are determined based on the absence of microbial growth on agar plates at varying drug concentrations. Although both *B. subtilis* and *B. cereus* exhibited an MBC of 100 mg/mL, the MBC/MIC ratio was higher for *B. cereus* (8) than for *B. subtilis* (4). These results suggest that the crude extract of MOLEF 4 exhibits a greater bactericidal tendency against *B. subtilis* compared to *B. cereus*.

The current findings suggest that the crude extract from *X. cubensis* exhibits antibacterial activity against both *Bacillus* species, with greater potency against *B. subtilis* at a lower MIC (12.50 mg/mL). To further investigate its effects, morphological changes in *B. subtilis* were examined using SEM, comparing untreated and treated cells. Treatment with the extract resulted in smaller bacterial cells relative to the control, indicating suppressed growth. Moreover, the extract caused perforation and disruption of the bacterial cell membranes, as evidenced by shrunken and lysed cells. It is worth noting that similar effects have been reported by El-Sherbiny *et al*. (2024) and Wizrah *et al*. (2026) using *Moringa* extracts, although these studies did not involve endophytic fungi. Most of the available micrographic evidence has focused on the effects of *Moringa* extracts on Gram-negative bacteria. For example, El-Sherbiny *et al*. (2024) demonstrated that *M. oleifera* extract caused irregular cell shapes, ruptured cell walls and membranes, and reduced cytoplasm density in *Klebsiella pneumoniae* and *E. coli*. Likewise, Wizrah *et al*. (2026) found that *Moringa peregrina* extract induced surface collapse, deep membrane perforation, irregular cavities, wall rupture and leakage of cytoplasmic contents in *Shigella sonnei*. The present and previous findings suggest that *Moringa* extracts and endophytes inhabiting the leaves mediate antibacterial activity primarily through bacterial cell membrane disruption. Generally, bacterial cell membranes are essential for survival, maintaining structural integrity, regulating nutrient transport and serving as key virulence factors, such as mediating adhesion to host cells during infection (Mohapatra & Arenas 2023).

Area normalisation is a quantitative approach used in GC–MS analysis to measure compound concentration in samples which suggests the relative abundance of specific compounds present at elevated levels within the crude extract. The crude extract of the isolated endophytic *X. cubensis* contains a mixture of the various compounds with potential antimicrobial activities and its concentration possibly affecting the efficacy of the antimicrobial properties.

Among the 42 constituents detected in the extract, four were classified as major compounds, which are assumed to play significant roles in mediating its therapeutic effects. The most abundant compound, representing the highest normalised area, was 2,4-DTBP. This compound is a widespread constituent of volatile or essential oils and has been reported in bacteria, fungi, diatoms, liverworts, pteridophytes, gymnosperms, dicots, monocots and animals. Interestingly, 2,4-DTBP exhibits notable toxicity toward various organisms, including its producers; however, the biological rationale for the production of potentially autotoxic 2,4-DTBP and its analogs remains unclear (Zhao *et al*. 2020). Early studies on 2,4-DTBP include Choi *et al*. (2013), who reported that 2,4-DTBP extracted from sweet potato protected pheochromocytoma (PC12) cells against hydrogen peroxide-induced oxidative stress and cytotoxicity. Consistent with these findings, sweet potato extract containing 2,4-DTBP reduced neuronal damage induced by amyloid-β (Aβ1–24) in a mouse model. This neuroprotective effect is likely mediated by the antioxidant capacity of 2,4-DTBP, which can scavenge reactive oxygen species and mitigate oxidative stress-related cellular damage. These studies highlight the potential of dietary or pharmacological sources of 2,4-DTBP, such as sweet potato, to inhibit or slow oxidative stress-induced disorders, including neurodegenerative diseases like Alzheimer’s disease. Importantly, the redox activity of 2,4-DTBP may also be relevant in microbial systems. In bacteria, disruption of oxidative homeostasis can lead to ROS accumulation, damaging cellular components and resulting in cell death. This study provides important insights into the potential of 2,4-DTBP as an antimicrobial agent, suggesting that its ability to perturb bacterial oxidative balance may contribute to its observed antibacterial effects.

The antimicrobial activities of 2,4-DTBP have been reported by Dharni *et al*. (2014), Varsha *et al*. (2015), Mishra *et al*. (2020), Kushveer *et al*. (2023) and Sundar and Arunachalam (2025). Dharni *et al*. (2014), Varsha *et al*. (2015) and Kushveer *et al*. (2023) demonstrated its antifungal activity when isolated from *Pseudomonas monteilii*, *Lactococcus* sp. and *Marasmiellus* sp., respectively. Mishra *et al*. (2020) showed that 2,4-DTBP, isolated from endophytic fungi such as *Daldinia eschscholtzii* (from *Tridax procumbens*), exhibits potent anti-quorum-sensing and anti-virulence activity against *P. aeruginosa*, attenuating biofilm formation, suppressing virulence factor production and downregulating key quorum-sensing genes (*lasI, lasR, rhlI* and *rhlR*). The compound also impaired bacterial adhesion and invasion of A549 lung epithelial cells displayed synergistic bactericidal activity with ampicillin, and was predicted computationally to bind quorum-sensing receptors LasR and RhlR. Sundar and Arunachalam (2025) reported that 2,4-DTBP, isolated from endophytic *Fusarium oxysporum*, exhibited strong activity against human pathogens. This included MRSA and vancomycin-resistant *Enterococcus faecalis*, with time-kill kinetics indicating a concentration- and time-dependent bactericidal effect. However, there is limited information on the effects of 2,4-DTBP against *B. subtilis*, and its mechanism of action in this organism remains unexplored.

To the best of our knowledge, this study is the first to report the isolation of 2,4-DTBP from *X. cubensis*. This is supported by Zhao *et al*. (2020), who noted that only 8 fungal families and 11 species are known to produce 2,4-DTBP, with *Xylaria* and the family Xylariaceae not previously documented. Moreover, a review by Chen *et al*. (2024), summarising compounds identified from 1994 to January 2024, indicated that none of the 445 newly reported compounds, including terpenoids, nitrogen-containing compounds, polyketides, lactones and other chemical classes, from *Xylaria* species corresponded to 2,4-DTBP. These findings highlight the novelty of its discovery in *X. cubensis* and underscore the potential value of further investigation, including *in vitro* and *in silico* studies, to elucidate its bioactivity and mechanistic properties.

Pyrrolo[1,2-a]pyrazine-1,4-dione, hexahydro-3-(1-methylethyl), has been reported as a potent antioxidant compound (Ser *et al*. 2015). Notably, antioxidant properties are often closely associated with antimicrobial activity. This relationship is supported by findings from Kiran *et al*. (2018), who demonstrated that pyrrolo[1,2-a]pyrazine-1,4-dione, hexahydro-isolated from the marine bacterium *Bacillus tequilensis*, effectively inhibited the growth of multidrug-resistant *S. aureus*. Similarly, Vikram and Hariram (2025) reported that GC–MS analysis of epoxy degradation products revealed a dominant contribution (98%) from pyrrolo[1,2-a]pyrazine-1,4-dione, hexahydro-, which exhibited significant anticandidal activity against *C. albicans*. These microorganisms are also well known for their ability to form biofilms, which arise from complex social interactions and represent an important survival strategy for many microbial species. Notably, pyrrolo[1,2-a]pyrazine-1,4-dione, hexahydro-has been reported to interfere with biofilm formation (Rajivgandhi *et al*. 2018; Abdulrahman *et al*. 2022). Rajivgandhi *et al*. (2018) demonstrated that this compound inhibited bacterial biofilm formation and significantly reduced the viability of preformed biofilms in *Proteus mirabilis* and *E. coli*. In addition, Abdulrahman *et al*. (2022) analysed secondary metabolites extracted from endophytic *Bacillus* sp., identifying pyrrolo[1,2-a]pyrazine-1,4-dione, hexahydro- as one of the major constituents. The extract exhibited strong antibiofilm activity against *Vibrio harveyi*, *Pseudoalteromonas shioyasakiensis* and *Planomicrobium* sp., with biofilm inhibition reaching up to 98.41%. Most of the aforementioned studies reported pyrrolo[1,2-a]pyrazine-1,4-dione, hexahydro- as a metabolite isolated from *Bacillus* species. Interestingly, this observation contrasted with our findings, in which the compound appeared to be associated with the inhibition of the bacteria. This apparent contradiction highlighted the need for further investigation into the underlying mechanisms by which pyrrolo[1,2-a]pyrazine-1,4-dione, hexahydro-may exert inhibitory effects.

A major compound in the crude extract of the endophytic fungus *X. cubensis* is 3-furanacetic acid, 4-hexyl-2,5-dihydro-2,5-dioxo-, a cyclic dicarboxylic anhydride. Although information on its antimicrobial activity is limited, similar compounds have been linked to notable bioactivities. Gemantari *et al*. (2021) reported that an ethyl acetate extract of *Eutypa linearis* from *Coleus amboinicus* contained this compound as a major constituent (74% GC-MS peak area) and exhibited antioxidant and cytotoxic activities. Likewise, Wang (2022) found that mango kernel oil from Jinhuang mango, containing the compound as a major component (7.3%), demonstrated antioxidant and antiproliferative effects. More recently, Mohammed *et al*. (2025) showed that it protected against cadmium chloride-induced oxidative damage in rats in a dose-dependent manner. Collectively, these findings suggest that 3-furanacetic acid, 4-hexyl-2,5-dihydro-2,5-dioxo-may contribute to the antioxidant and cytoprotective properties of fungal extracts, which are often linked to antimicrobial activity.

1,3,5-Xylenol, also known as 3,5-dimethylphenol, is a phenolic compound structurally derived from m-xylene. Similar to 3-furanacetic acid, 4-hexyl-2,5-dihydro-2,5-dioxo-, limited information is available regarding its antimicrobial activity. According to Yao *et al*. (2023), 1,3,5-xylenol serves as a precursor to more potent related antimicrobials, such as 4-chloro-3,5-dimethylphenol (PCMX), which is widely used in the production of pesticides, disinfectants and preservatives. Certain bacteria, including *Pseudomonas* sp. (Chapman 1972) and *Rhodococcus* sp. (Yao *et al*. 2023), are capable of metabolizing 3,5-xylenol. Earlier studies by Chapman (1972) suggested that 1,3,5-xylenol is eventually converted into 3-hydroxybenzoate, although the precise pathway remained unclear. More recent work by Yao *et al*. (2023) demonstrated that 1,3,5-xylenol is converted to 3-hydroxy-5-methylbenzoate and subsequently to 5-hydroxyisophthalate, which then enters the protocatechuate pathway, rather than producing 3-hydroxybenzoate. In relation to our study, 1,3,5-xylenol may assist the antimicrobial compounds present in the *X. cubensis* extract by acting as a precursor, and its exact role and metabolic pathway merit further investigation.

## CONCLUSION

The ethyl acetate extract from an endophytic fungus closely related to *X. cubensis*, isolated from *M. oleifera* leaves, demonstrated strong antibacterial activity against *B. subtilis*, likely through disruption of the bacterial cell membrane. Ethyl acetate was selected as the extraction solvent due to its broad applicability in secondary metabolite studies, although future work should consider alternative solvents tailored to specific metabolite classes. Further studies are needed to evaluate the safety of the extract and advanced approaches such as multi-omics could help clarify its mechanisms and enhance its therapeutic potential. A limitation of this study is that only mature leaves were used, which may have influenced the diversity of endophytic fungi compared to younger tissues. In addition, antibacterial activity was assessed only against *Bacillus* species, and the GC–MS analysis provided a preliminary chemical profile without bioassay-guided fractionation. These limitations highlight the need for broader microbial testing, fractionation studies, and evaluation of leaf age effects in future research.

## Figures and Tables

**FIGURE 1:**
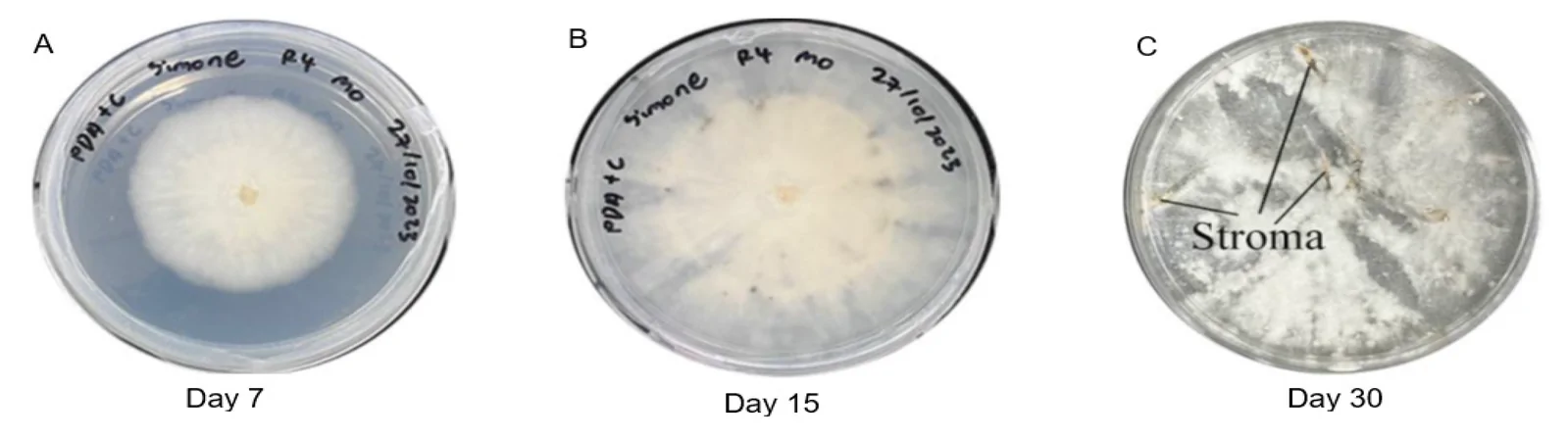
Macroscopic morphology of MOLEF 4 grown on PDA at (A) day 7; (B) day 15; and (C) day 30, showing progressive mycelial expansion, increased aerial hyphae density and stromatic structure formation at later stages.

**FIGURE 2:**
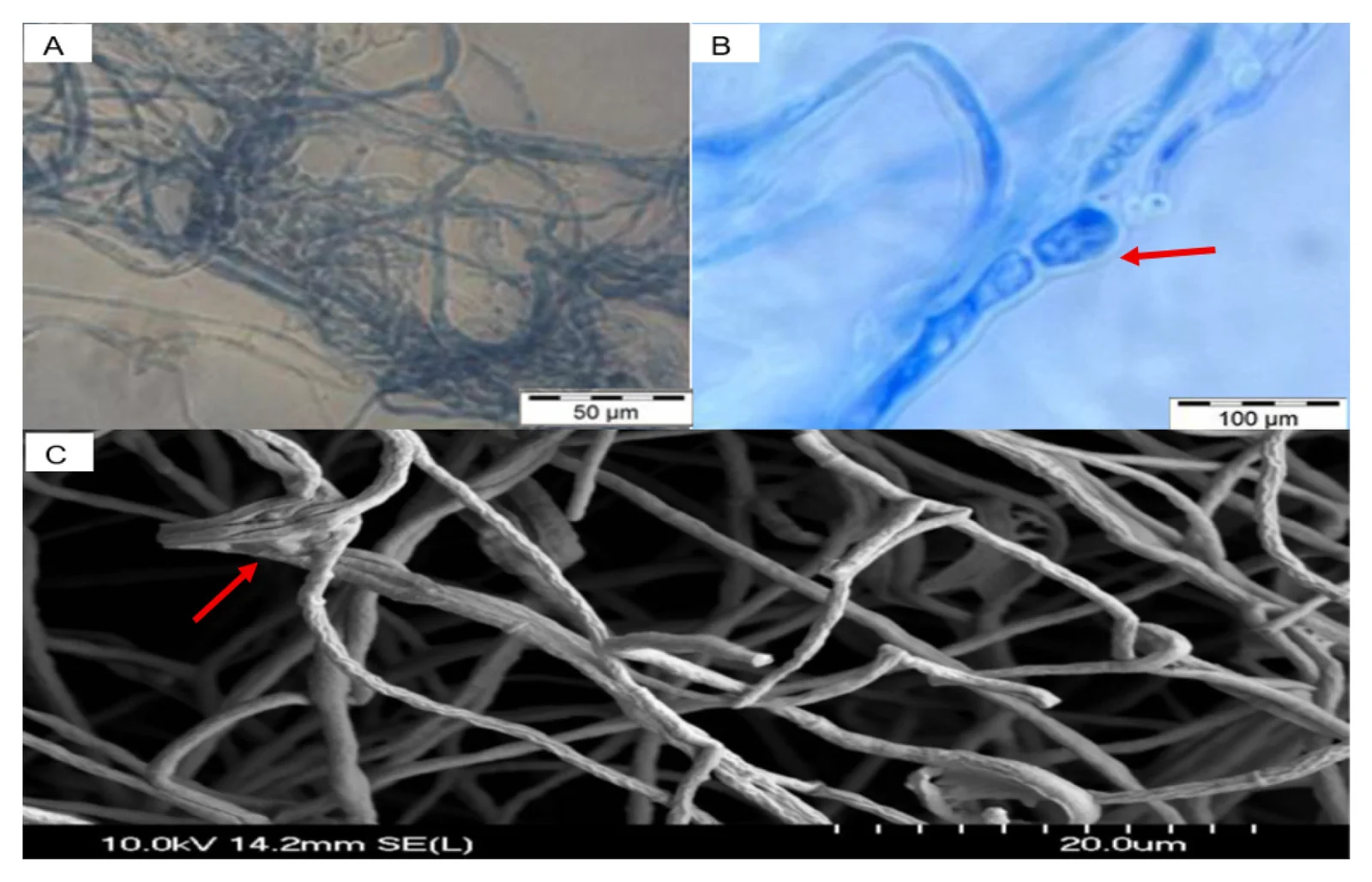
Microscopic observations of MOLEF 4 using (A–B) light microscopy; and (C) scanning electron microscopy (SEM). Overlapping mycelia and segmented hyphae were observed at low magnification (4×) (A). At higher magnification (40×), the segmented hyphae were more clearly defined, and the presence of swollen hyphae or conidia-like structure was evident (red arrow). The occurrence of these swollen hyphae was further confirmed by SEM analysis.

**FIGURE 3:**
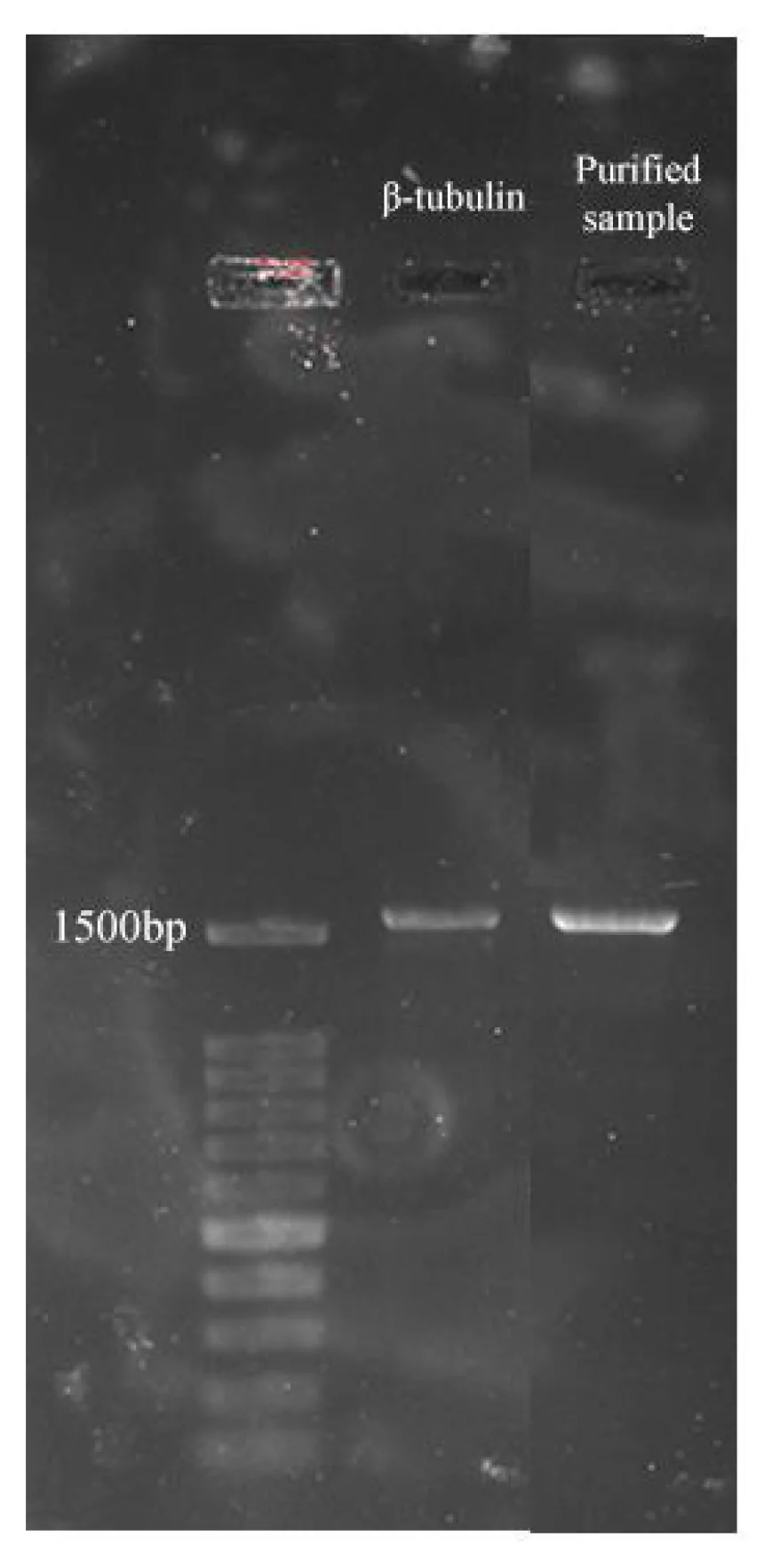
Gel electrophoresis of the fungal isolate MOLEF4 showing PCR amplification of the β-tubulin (tub2) gene using gene-specific primers T1 and T22 for molecular identification.

**FIGURE 4:**
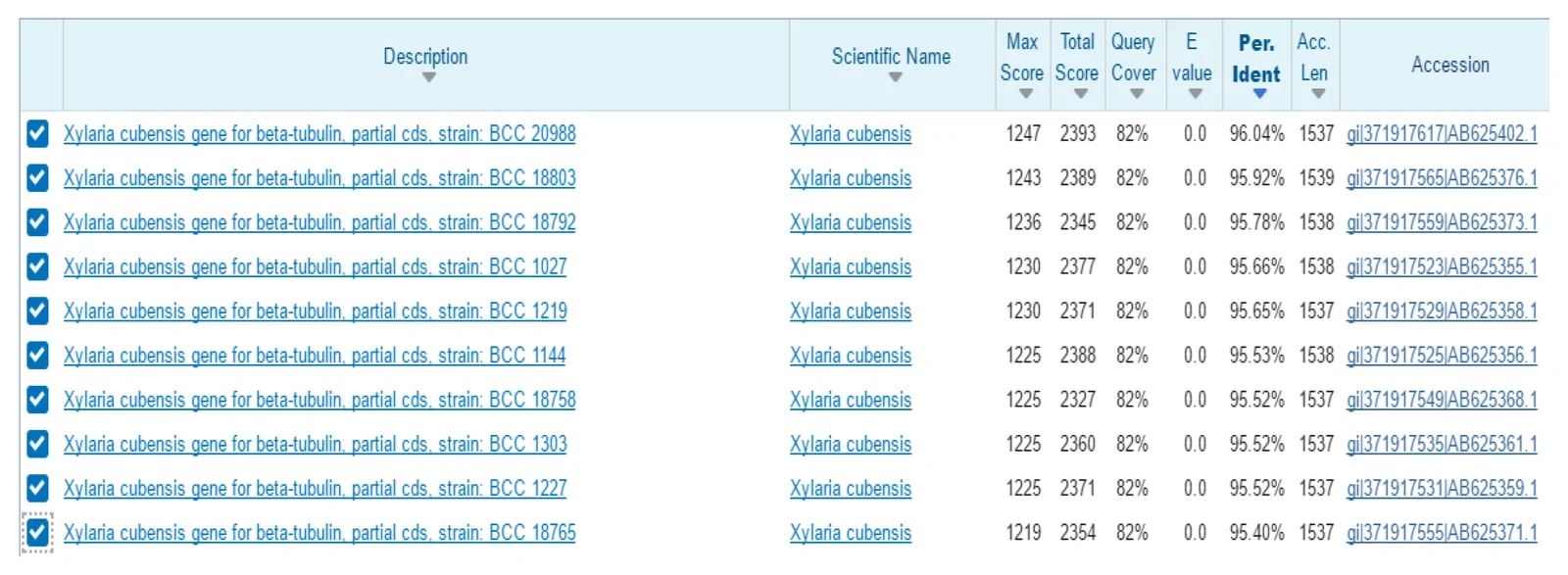
BLAST analysis showing > 95% sequence similarity between MOLEF 4 and *X. cubensis*.

**FIGURE 5:**
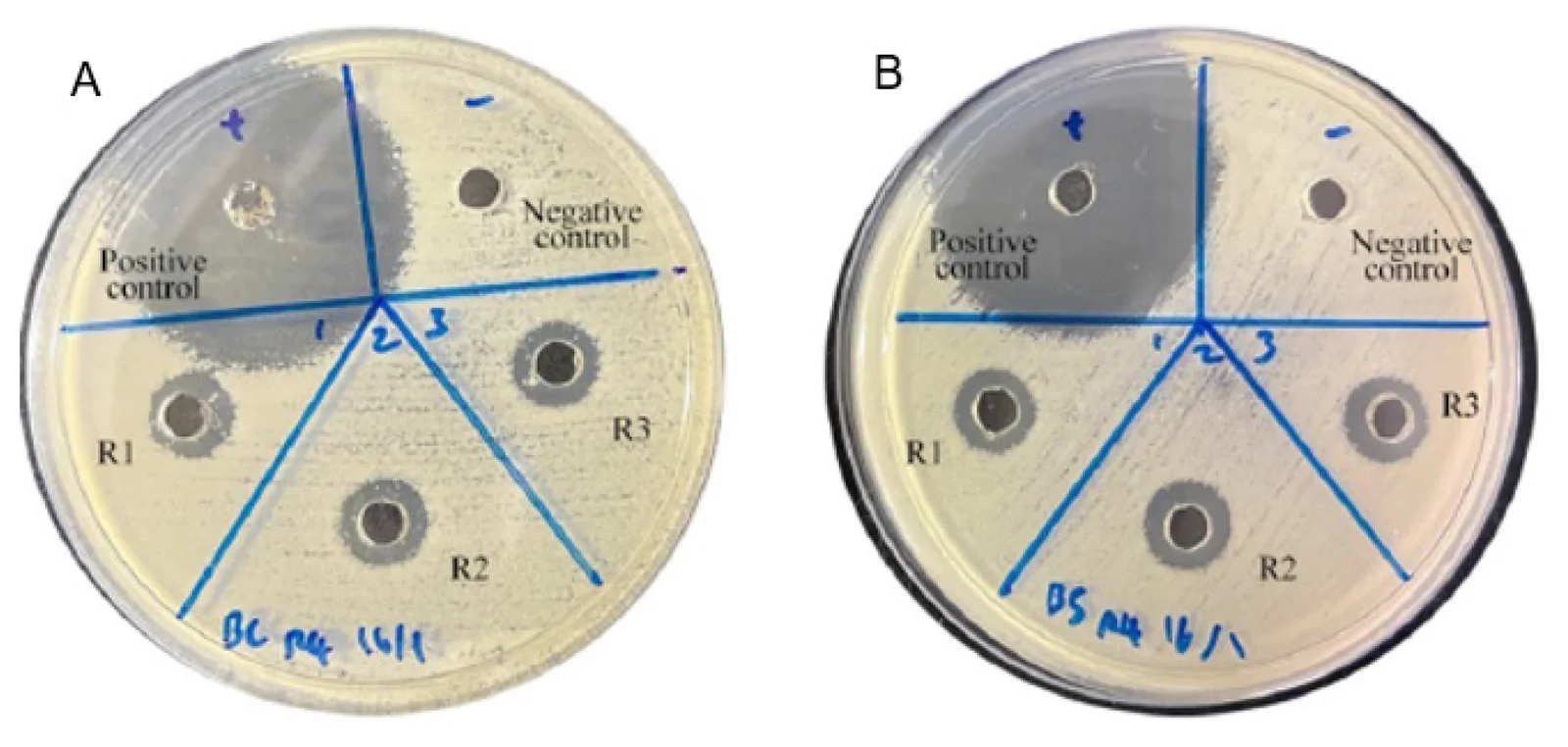
Antibacterial activity of *X. cubensis* ethyl acetate extract (100 mg/mL) against (A) *B. cereus*; and (B) *B. subtilis* assessed by well diffusion. Chloramphenicol and 25% methanol served as positive and negative controls, respectively.

**FIGURE 6:**
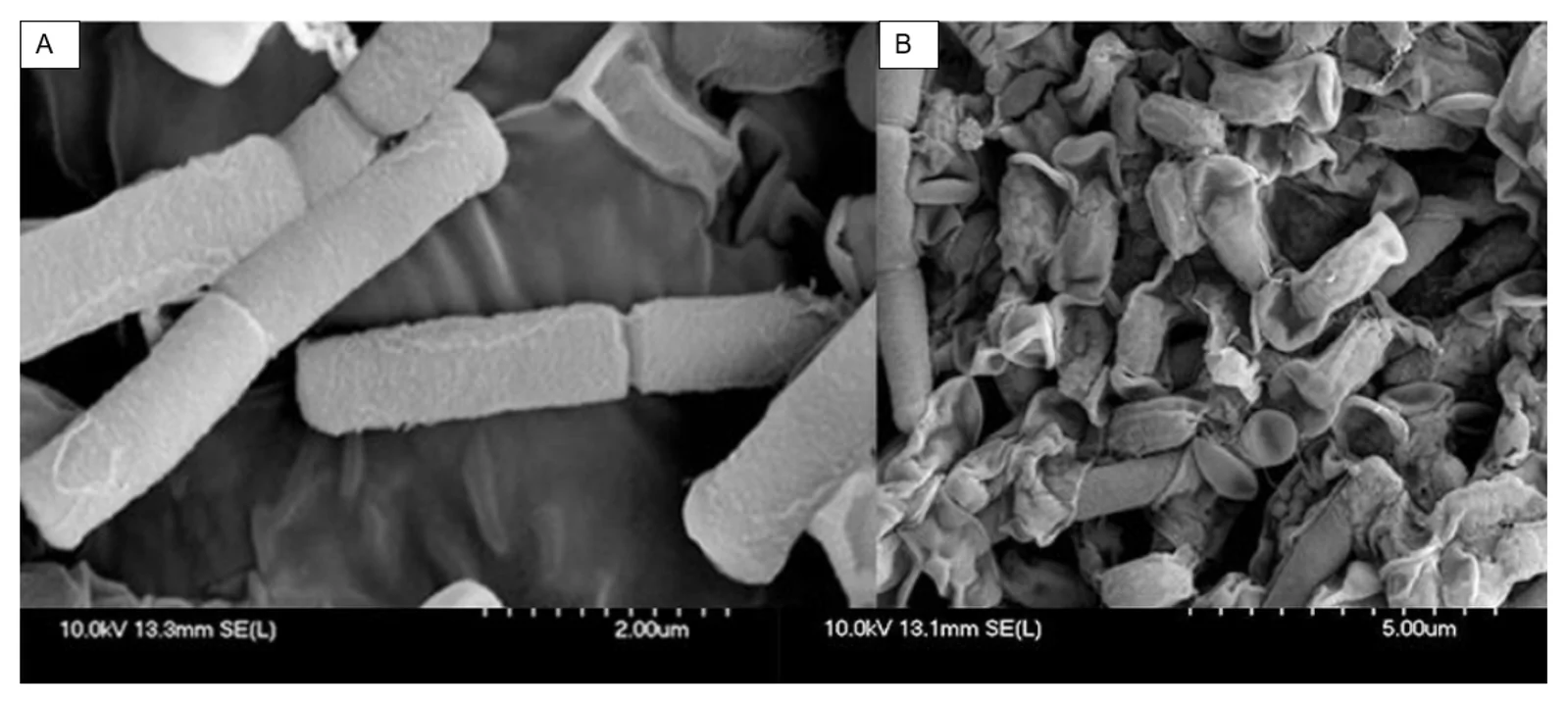
SEM images of *B. subtilis* (A) before treatment; and (B) after treatment with the ethyl acetate extract of *X. cubensis*. Untreated cells displayed normal rod-shaped morphology, whereas cells exposed to 100 mg/mL extract (MBC) showed cell shrinkage and lysis.

**FIGURE 7:**
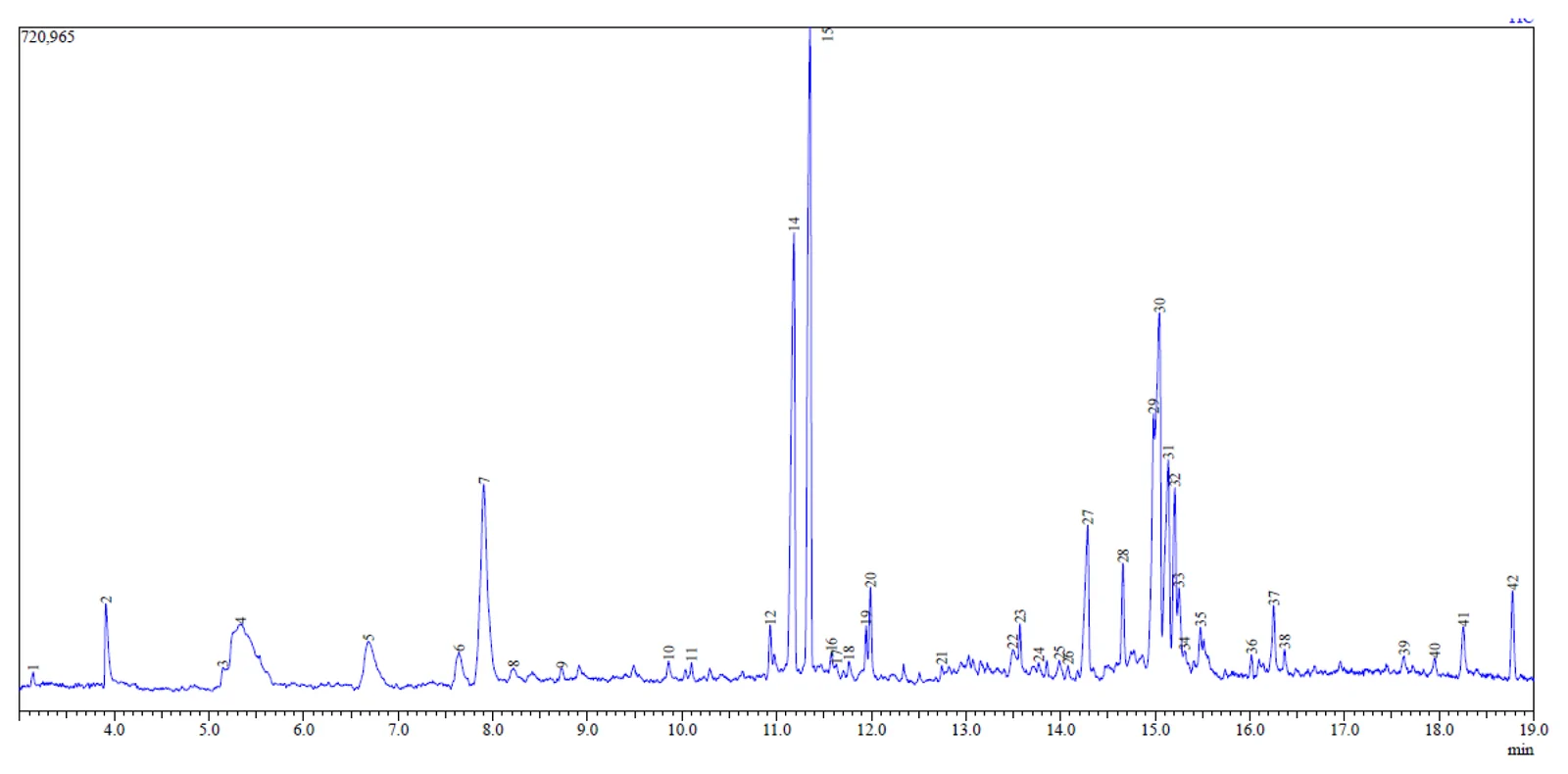
GC–MS analysis revealed a total of 42 peaks within a retention time of 19 min. Four major compounds were detected at retention times of 7.902 min, 11.182 min, 11.351 min and 15.045 min, corresponding to 1,3,5-xylenol; 3-furanacetic acid, 4-hexyl-2,5-dihydro-2,5-dioxo-; 2,4-di-tert-butylphenol (2,4-DTBP); and pyrrolo[1,2-a]pyrazine-1,4-dione, hexahydro-3-(1-methylethyl), respectively.

**TABLE 1:** Isolate MOLEF 4 exhibited inhibitory activity against six of the seven pathogenic microorganisms evaluated in the agar plug assay.

Fungal isolate	Mean of inhibition zone diameter against test microorganisms (mm ± standard deviation)						

	*B. cereus*	*B. subtilis*	*E. coli*	*P. aeruginosa*	*S. Typhimurium*	*S. aureus*	*C. albicans*
MOLEF 1	-	-	-	-	11.0 ± 1.0	-	-
MOLEF 2	-	-	-	-	9.3 ± 2.3	-	-
MOLEF 3	-	-	-	-	-	-	-
MOLEF 4	10.3 ± 1.2	9.7 ± 0.5	8.2 ± 0.8	9.8 ± 0.3	8.5 ± 1.3	8.8 ± 0.3	-
MOLEF 5	-	-	-	-	10.0 ± 1.7	-	-
MOLEF 6	-	-	-	10.0 ± 1.0	-	10.0 ± 2.0	-
MOLEF 7	9.3 ± 0.6	-	9.2 ± 1.4	8.2 ± 0.3	-	8.5 ± 0.5	-

**TABLE 2 t2-tlsr-37-3-181:** Quantitative antimicrobial assessment of the ethyl acetate extract of *X.cubensis* using well diffusion and broth microdilution assays.

Test microorganism	Diameter of inhibition zone (mm)	MIC value (mg/mL)	MBC value (mg/mL)	MBC/MIC	Biological effect
*B. cereus*	10.7 ± 0.5	12.5	100	8	Bacteriostatic
*B. subtilis*	10.3 ± 0.5	25	100	4	Bactericidal
*E. coli*	-	-	-	-	-
*P. aeruginosa*	-	-	-	-	-
*S. Typhimurium*	-	-	-	-	-
*S. aureus*	-	-	-	-	-
*C. albicans*	-	-	-	-	-

**TABLE 3:** GC–MS-identified compounds from the ethyl acetate extract of *X. cubensis*, highlighting major constituents (> 10% area normalisation).

No.	Compound name	Retention time	m/z	Area	Height	Area normalisation (%)
1	Acetamide	3.141	59.00	30576	13648	0.970
2	Sclerosol	3.910	63.00	135591	38405	4.303
3	Phenyl alcohol	5.146	94.00	41282	9086	1.310
4	Phenyl alcohol	5.349	94.00	168137	33175	5.336
5	Phenol, 3-methyl-	6.690	108.00	119273	13408	3.785
6	Phenol, 2,5-dimethyl-	7.643	122.00	39898	7627	1.266
7	Xylenol <1,3,5->	7.902	107.00	324081	55690	10.285
8	Phenol, 3,4-dimethyl-	8.207	107.00	18197	3843	0.577
9	2-Acetyl-3-methylthiophene	8.721	125.00	7634	3053	0.242
10	Phenol, 4-(2-propenyl)-	9.858	134.00	7933	3197	0.252
11	1-Pentadecene	10.099	55.00	3756	2009	0.119
12	Dimethyl phthalate	10.931	163.00	50614	25265	1.606
13	2,5-Cyclohexadiene-1,4-dione, 2,6-bis(1,1-d	-	177.00	-	-	D.(Ref)
14	3-Furanacetic acid, 4-hexyl-2,5-dihydro-2,5	11.182	126.00	333226	120719	10.575
15	2,4-Di-tert-butylphenol	11.351	191.00	606771	245160	19.256
16	Acetamide, N-(2-phenylethyl)-	11.578	104.00	21967	11351	0.697
17	2-Aminooctane, N-acetyl-	11.631	86.00	4526	2731	0.144
18	Dodecanoic acid	11.764	73.00	2478	1574	0.079
19	Octadec-1-ene	11.945	83.00	6831	5086	0.217
20	1H-2-Benzopyran-1-one, 3,4-dihydro-8-hyd	11.989	178.00	23781	13833	0.755
21	Pentafluoropropionic acid, dodecyl ester	12.744	69.00	2038	1149	0.065
22	1,4-diazabicyclo[4.3.0]nonan-2,5-dione, 3-m	13.497	70.00	23448	6384	0.744
23	Octadec-1-ene	13.571	83.00	6382	4471	0.203
24	Chamigrene <beta->	13.770	189.00	1625	957	0.052
25	3-Chloropropionic acid, heptadecyl ester	13.990	114.00	4871	1836	0.155
26	3-Isopropyl-2,5-piperazine-dione	14.079	114.00	16184	6688	0.514
27	Cyclo(L-prolyl-L-valine)	14.286	154.00	113105	37467	3.589
28	7,9-Di-tert-butyl-1-oxaspiro(4,5)deca-6,9-di	14.660	57.00	15183	9563	0.482
29	Hexadecanoic acid <n->	14.980	73.00	15020	10591	0.477
30	Pyrrolo[1,2-a]pyrazine-1,4-dione, hexahydr	15.045	154.00	533582	113571	16.933
31	Pyrrolo[1,2-a]pyrazine-1,4-dione, hexahydr	15.137	154.00	187473	66921	5.949
32	3,5-di-tert-Butyl-4-hydroxyphenylpropionic	15.209	263.00	86626	41562	2.749
33	Pyrrolo[1,2-a]pyrazine-1,4-dione, hexahydr	15.254	70.00	55174	21824	1.751
34	Methyl octadec-6,9-dien-12-ynoate	15.314	91.00	4769	1925	0.151
35	2H-Pyrrol-2-one, 1,5-dihydro-4-(1-pyrrolidi	15.477	152.00	26444	13857	0.839
36	RT:16.017	16.016	154.00	4242	2455	0.135
37	RT:16.250	16.249	167.00	10676	4552	0.339
38	Eicosene	16.368	57.00	5420	2460	0.172
39	Octacosyl trifluoroacetate	17.624	57.00	4498	1823	0.143
40	Pyrrolo[1,2-a]pyrazine-1,4-dione, hexahydr	17.953	125.00	8825	3229	0.280
41	Pyrrolo[1,2-a]pyrazine-1,4-dione, hexahydr	18.255	125.00	25351	10592	0.805
42	Phthalic acid, bis(2-ethylhexyl) ester (6CI,8	18.776	149.00	53620	26718	1.702
